# Targeting Neuroplasticity in Substance Use Disorders: Implications for Therapeutics

**DOI:** 10.1146/annurev-pharmtox-061724-080548

**Published:** 2024-12-17

**Authors:** Marina E. Wolf

**Affiliations:** Department of Behavioral Neuroscience, Oregon Health & Science University, Portland, Oregon, USA

**Keywords:** glutamate, nucleus accumbens, psychostimulants, substance use disorder, synaptic plasticity, therapeutic approaches

## Abstract

The last two decades have witnessed substantial advances in identifying synaptic plasticity responsible for behavioral changes in animal models of substance use disorder. We have learned the most about cocaine-induced plasticity in the nucleus accumbens and its relationship to cocaine seeking, so that is the focus in this review. Synaptic plasticity pointing to potential therapeutic targets has been identified mainly using two drug self-administration models: extinction-reinstatement and abstinence models. A relationship between cocaine seeking and potentiated AMPAR transmission in nucleus accumbens is indicated by both models. In particular, an atypical subpopulation—Ca^2+^-permeable or CP-AMPARs—mediates cue-induced seeking that persists even after long periods of abstinence, modeling the persistent vulnerability to relapse that represents a major challenge in treating substance use disorder. We review strategies to reverse CP-AMPAR plasticity; strategies targeting other components of excitatory synapses, including dysregulated glutamate uptake and release; and behavioral interventions that can be augmented by harnessing synaptic plasticity.

## 1. INTRODUCTION AND SCOPE

Substance use disorder (SUD) is a global problem. A major challenge for recovering users is avoiding relapse to drug taking (1). The persistence of relapse vulnerability suggests that drug misuse triggers long-lasting alterations in brain circuits processing motivated behavior. Thus, tremendous effort has been expended to identify the synaptic plasticity in these circuits that maintains vulnerability to relapse. This review discusses therapeutic approaches suggested by such studies. Areas of focus are described below:

1. The article concentrates on psychostimulants (mainly cocaine) because we know the most about psychostimulant-induced plasticity. Stimulant misuse remains a significant problem for the United States (2–6). There is no US Food and Drug Administration (FDA)-approved pharmacotherapy for cocaine or methamphetamine use disorder. The standard of care is behavioral intervention (3).
2. Most synaptic plasticity studies in addiction models have been performed in the nucleus accumbens (NAc), a key gateway for addiction-related behavior, so this region is our focus. In particular, we review studies of medium spiny neurons (MSNs), the principal neurons of the NAc. Consideration of this literature is sufficient to capture both the promise and pitfalls of developing therapeutics based on synaptic plasticity research. Plasticity in many other regions certainly contributes to SUD. However, from a practical standpoint, potential therapeutic approaches will affect the whole brain (pharmacological) or NAc-related circuits (brain stimulation) and will therefore have to contend with plasticity in the NAc. The role of nonneuronal cells in this plasticity has been reviewed elsewhere (7).
3. Drug self-administration models are emphasized, as they are unique in enabling assessment of motivation to use drugs. In these models, as for humans, drug seeking can be triggered by cues/contexts previously paired with the drug, a stressor, or a priming injection of drug. We focus here on cue-induced seeking. The complexity of stress-induced drug seeking is beyond our scope (8), and drug reexposure produces brain effects that complicate the identification of plasticity underlying relapse-like behavior.
4. The article focuses on therapeutic targets that are related to components of excitatory synapses and have been identified at least in part by studying synaptic transmission; for a more general review of therapeutic approaches, the reader is referred elsewhere (9–12).

We begin with an overview of components of the glutamate synapse and their relevance to SUD. Then, we review therapeutically relevant plasticity identified by two major self-administration models and assess the potential of targets identified by this work. More detail is provided for abstinence models because extinction-reinstatement models have been more thoroughly covered by prior reviews.

## 2. COMPONENTS OF THE GLUTAMATE SYNAPSE AND OVERVIEW OF ADDICTION RELEVANCE

### 2.1. AMPA Receptors

AMPA receptors (AMPARs) are ionotropic glutamate receptors comprised of GluA1–4 subunits and are regulated at the levels of expression, posttranslational modification, and trafficking (13). They are responsible for the majority of excitatory synaptic transmission, and many studies have demonstrated a necessary role for AMPAR transmission in MSNs of the NAc in drug seeking (14). However, given their central role in every brain process, administering pan-AMPAR antagonists to treat SUD is problematic. A more viable approach is to target an atypical AMPAR population that can be selectively manipulated. In drug-naïve animals, principal neurons in most brain regions, including NAc MSNs, express mainly Ca^2+^-impermeable AMPARs (CI-AMPARs) (15, 16). A less common subtype, Ca^2+^-permeable AMPARs (CP-AMPARs), exhibits larger single-channel conductance and faster kinetics, and displays voltage-dependent block by intracellular polyamines resulting in an inwardly rectifying current-voltage relationship (quantified as the rectification index); as a result of these properties, CP-AMPAR insertion strengthens synapses and enables them to exhibit distinct forms of plasticity (17). For an example of altered plasticity related to cocaine, see Mameli et al. (18). Cocaine-induced elevation of CP-AMPARs was first discovered in ventral tegmental area (VTA) dopamine neurons (19, 20). Soon afterward, it was shown that CP-AMPAR upregulation in excitatory synapses on nucleus accumbens core (NAcC) MSNs is required for expression of incubated cocaine craving (16). Extensions of this work (Section 5) suggest potential therapeutic strategies aimed at reducing CP-AMPAR transmission.

### 2.2. NMDA Receptors

*N*-methyl-d-aspartate receptors (NMDARs) are tetramers comprised of two obligatory GluN1 subunits along with GluN2 (GluN2A-D) and/or GluN3 (GluN3A or GluN3B) subunits. They can be diheteromeric receptors (e.g., GluN1/GluN2A) or triheteromeric receptors that contain either two different GluN2 subunits or a combination of GluN2 and GluN3 subunits (21). Excitatory transmission at near resting membrane potentials is typically mediated by AMPARs because NMDARs are blocked at hyperpolarized potentials by Mg^2+^. However, under conditions where glutamate is bound and the postsynaptic membrane is depolarized, the NMDAR channel opens to enable Ca^2+^ entry. Through this coincidence detection, NMDARs are vital to many forms of synaptic plasticity, collectively called NMDAR-dependent plasticity. The demonstration that NMDAR activation is required for behavioral sensitization was one of the first lines of evidence supporting a role for synaptic plasticity in addiction (22, 23). Complex NMDAR plasticity in addiction models has subsequently been revealed (24). While NMDAR antagonists can be associated with unacceptable side effects, work is underway to optimize a new generation of NMDAR modulators (25). An atypical NMDAR subtype contains the GluN3 subunit (26). Genetic studies indicate GluN3 association with SUD phenotypes (27), and GluN3-containing NMDARs are associated with CP-AMPAR plasticity in animal models (Section 5.1), suggesting potential therapeutic utility of their selective targeting.

### 2.3. Kainate Receptors

Kainate receptors (GRIK1–5 subunits) modulate transmitter release presynaptically and mediate synaptic transmission postsynaptically. While they are increasingly implicated in brain disorders, including alcohol use disorder (28), elucidating their role in addiction-related plasticity will require more selective pharmacological tools.

### 2.4. Group I Metabotropic Glutamate Receptors (mGlu1 and mGlu5)

mGlu1 and mGlu5 are postsynaptic receptors generally located in a perisynaptic zone surrounding ionotropic glutamate receptors, making them well positioned to regulate effects of ionotropic glutamate receptors as well as ion channels at postsynaptic sites (29, 30). They are best known for mediating diverse types of long-term depression (LTD) through multiple signaling pathways, including canonical coupling to Gα_q/11_ leading to Ca^2+^ mobilization from intracellular stores (31). Orthosteric agonists and antagonists exist for each receptor, as well as positive and negative allosteric modulators (PAMs and NAMs). The latter offer advantages as therapeutic agents due to the greater ability of allosteric modulation to differentiate between metabotropic glutamate receptor (mGluR) subtypes, bias particular mGluRs toward conformations that selectively activate a particular signaling pathway, and selectively influence synapses being activated by endogenous glutamate (32). Most work in addiction models has focused on mGlu5 NAMs (Section 4), but mGlu1 is emerging as a target for craving reduction due to its ability to internalize CP-AMPARs (Section 5).

### 2.5. Group II Metabotropic Glutamate Receptors (mGlu2, mGlu3) and Group III Metabotropic Glutamate Receptors (mGlu4, mGlu6, mGlu7, mGlu8)

As reviewed recently (30), these mGluRs couple to Gα_i/o_ to inhibit adenylyl cyclase, although they also influence other effectors. They are expressed primarily on nerve terminals and depress glutamate release in NAc and other regions. mGlu3 is expressed both pre- and postsynaptically, as well as on glia, and accordingly is implicated in multiple forms of plasticity. Pharmacological activation of Group II mGluRs reduces seeking of cocaine and other drugs in both reinstatement and abstinence models by reducing glutamate release (33, 34). This effect is likely attributable to mGlu2; however, mGlu3 is also a promising target for SUD and other disorders (35). mGlu7 activation may also reduce drug seeking (34).

## 3. EXTINCTION-REINSTATEMENT AND ABSTINENCE MODELS: OVERVIEW

In the extinction-reinstatement model, rats/mice self-administer drug until stable responding is established, typically using a limited-access regimen. Animals then undergo extinction training. After responding is extinguished, relapse is modeled using a reinstatement test.

In abstinence models, particularly those designed to elicit incubation of craving (see below), an extended-access regimen is commonly used. After self-administration is completed, animals undergo a period in which the drug is not available (termed withdrawal or abstinence). Relapse is then modeled by conducting a cue-induced seeking test. The number of operant responses during the test provides a measure of cue-induced seeking or craving.

Abstinence models were used to demonstrate a phenomenon termed incubation of craving (36). Incubation refers to the progressive increase in cue-induced responding over the first weeks of abstinence from drug self-administration; this is followed by a long plateau phase in which cue responsivity remains high before ultimately declining. Incubation has been demonstrated in rats after self-administration of cocaine, methamphetamine, opioids, nicotine, and alcohol (37). Translational relevance of this model is supported by reports of incubation in humans (see the sidebar titled Incubation of Craving in Humans) and by the persistence of heightened craving both in rats after incubation and in persons with SUD after long periods of abstinence.

For cocaine, which has been most extensively studied, extended-access self-administration regimens are more likely to elicit incubation of craving (36), although this is not absolute (38). Because of variability across regimens, incubation must be confirmed by comparing cue-induced seeking in early and late withdrawal. With regimens used to study NAc plasticity during cocaine incubation, elevated seeking is reliably observed after several weeks and remains high for months. To date, all studies of plasticity associated with incubation have been performed using forced abstinence (i.e., rats remain in home cages). Voluntary abstinence models have been developed and are used to mimic aspects of contingency management approaches in humans (39). In such models, plasticity would reflect not only drug self-administration and abstinence but also the effect of manipulations maintaining voluntary abstinence.

The plasticity and circuits recruited by extinction-reinstatement versus abstinence models differ in some respects (40). This is likely because extinction is a form of learning (the animal learns that the operant response no longer predicts drug delivery) that elicits its own plasticity. This complicates the disentanglement of plasticity resulting from drug self-administration, the abstinence period, and extinction training. Nevertheless, both models point to excessive/maladaptive glutamate transmission in the NAc as a driving force for relapse-like behavior, as detailed below. To the extent it has been examined, male and female rodents generally show similar cue-induced craving and relapse behavior (41).

## 4. EXTINCTION-REINSTATEMENT MODEL: THERAPUETICALLY RELEVANT PLASTICITY

The focus of this section is on work demonstrating a critical role for dysregulated glutamate transmission in the rat NAcC in the reinstatement of cocaine seeking.

### 4.1. Glutamate Adaptations After Completion of Extinction Training (No Relapse Test)

Extensive work supports a cascade in which cocaine self-administration followed by extinction decreases basal extracellular glutamate levels in the NAcC due to downregulation of the catalytic subunit (xCT) of the astrocytic cystine-glutamate exchanger (a major source of nonsynaptic extracellular glutamate in NAcC). This results in decreased tone on presynaptic mGlu2/3 receptors that inhibit glutamate release. In addition, mechanisms that normally restrict glutamate release to synapses are impaired due to reduced function of the astrocytic glutamate transporter GLT-1 (a major contributor to glutamate clearance) and retraction of astrocyte processes from MSN synapses. As a result, cue presentation during a reinstatement test produces augmented glutamate spillover that drives reinstatement of cocaine seeking by promoting the activation of postsynaptic AMPARs and mGlu5. This overall situation is referred to as disruption of glutamate homeostasis. Agents that restore glutamate homeostasis, such as *N*-acetylcysteine and ceftriaxone (Section 4.3), reduce reinstatement of cocaine seeking (9, 42–44). Some aspects of this cascade have been observed for other drugs of abuse following self-administration and extinction (9, 42–45).

Although the initial discovery of the above mechanisms relied largely on neurochemistry and behavior, conclusions were supported by electrophysiological studies (e.g., 46). Most studies that assessed AMPA/NMDA ratios in NAcC MSNs found increases after cocaine self-administration and extinction (46–48). Interestingly, in field recordings, evidence for the potentiation of excitatory transmission [greater field amplitude and long-term potentiation (LTP) occlusion] was found when cocaine self-administration was followed by 2–3 weeks of extinction or abstinence, whereas impaired LTD induction was found only after extinction training. Impairments in both LTP and LTD were reversed by *N*-acetylcysteine (49, 50).

Synaptic potentiation after forced abstinence from cocaine self-administration is sometimes due to CP-AMPAR insertion (Section 5). The only study to directly assess whether this also occurs after extinction found no difference in CP-AMPAR levels, using the rectification index, between nucleus accumbens shell (NAcS) MSNs in rats subjected to cocaine self-administration and extinction versus saline controls (NAcC was not evaluated); however, injection of the selective CP-AMPAR antagonist Naspm into NAcC or NAcS of identically treated animals reduced cocaine-primed reinstatement (51). Biotinylation studies found increased surface GluA1 but not GluA2 in NAcC after cocaine self-administration and extinction, and normalizing GluA1 by administering ceftriaxone blocked cue-induced reinstatement (52). Based on the abstinence literature (Section 5.1), it was suggested that ceftriaxone downregulates CP-AMPARs by elevating glutamate and thus activating mGlu1. Indeed, ceftriaxone inhibited the expression of incubated cue-induced craving after a cocaine self-administration regimen that would be predicted, based on other work, to depend on CP-AMPAR upregulation (53). Finally, in the cocaine conditioned place preference (CPP) model, CP-AMPARs accumulated in NAcS synapses after extinction training (54). If CP-AMPAR upregulation was prevented by administering ceftriaxone during extinction training, the threshold for CPP reinstatement was increased, suggesting that CP-AMPARs facilitate retrieval and expression of cocaine memories (55). Overall, these findings indicate that CP-AMPAR upregulation may occur when learning about cocaine-related cues is followed by either abstinence or extinction training, broadening its therapeutic relevance.

### 4.2. Transient Synaptic Potentiation During Relapse-Like Behavior

Remarkably, re-exposing rats to cocaine cues after extinction elicits an increase in spine head diameter and synaptic strength (AMPA/NMDA ratio) in NAcC MSNs that is detectable within 15 min but reverses within 45–120 min. This transient synaptic potentiation (TSP) correlates with the magnitude of reinstatement and is required for reinstatement (43, 47). More recent work in mice suggests that cue-induced cocaine seeking is associated with TSP in dopamine D1 receptor–expressing MSNs (D1 MSNs) after two to three weeks of extinction or abstinence, while refraining from seeking after extinction is associated with TSP in dopamine D2 receptor–expressing MSNs (D2 MSNs) (56). TSP also occurs during reinstatement of heroin (57) and nicotine (58) seeking.

The cascade leading to TSP results from glutamate spillover during reinstatement (Section 4.1). This glutamate activates mGlu5 on neuronal nitric oxide synthase–expressing interneurons, leading to nitric oxide synthesis and induction of matrix metalloprotease (MMP) activity. MMPs are a family of enzymes that modify extracellular matrix proteins to modulate synaptic plasticity. Transient activation of MMP-9 occurs during cue-induced reinstatement tests after self-administration of cocaine as well as nicotine and heroin, while MMP-2 activity is constitutively increased after cocaine self-administration and extinction (no reinstatement test); a constitutive increase in MMP activity (likely MMP-2) was also observed after nicotine self-administration and extinction (44, 48, 59). During abstinence from methamphetamine self-administration, MMP-2/9 activity remains unchanged until a seeking test is administered; however, while increased duration of abstinence was associated with incubation of methamphetamine seeking, the magnitude of MMP activation remained constant (60).

As some features of this cascade hold for several drugs of abuse but not sucrose, it has been hypothesized that the ability of drug-associated cues to transiently recruit additional neurons to a drug-memory ensemble (through TSP) accounts for the more potent ability of such cues to drive behavior compared to natural rewards that do not elicit TSP (43). However, while TSP occurs after both extinction and abstinence (see above), other abstinence studies have demonstrated an opposite form of rapid cue-evoked plasticity (Section 5.4).

### 4.3. Pharmacotherapies Suggested by Extinction-Reinstatement Studies

This section briefly describes strategies for reducing relapse-like behavior that have been identified in preclinical studies using the extinction-reinstatement model.

#### 4.3.1. Ceftriaxone.

As reviewed elsewhere (42), the antibiotic ceftriaxone restores glutamate homeostasis by normalizing the reductions in xCT and GLT-1 produced by cocaine self-administration and extinction. This normalizes glutamate transmission, preventing reinstatement. Some studies have found that ceftriaxone also reduces addiction-related behaviors for other drug classes, particularly opioids. Ceftriaxone also exerts beneficial effects in preclinical models of neurological disorders associated with excessive glutamate levels. As a therapeutic, ceftriaxone is well tolerated, but there is no orally bioavailable formulation. No clinical trials for ceftriaxone in SUD have been conducted.

#### 4.3.2. *N*-acetylcysteine.

*N*-acetylcysteine restores glutamate homeostasis by increasing activity of the cystine-glutamate exchanger and upregulating GLT-1, preventing glutamate spillover and reinstatement (9, 44). On the basis of robust results in preclinical models, *N*-acetylcysteine advanced to clinical trials in cocaine users. Results have been mixed, but some studies found reductions in craving (61). A double-blind placebo-controlled trial in methamphetamine-dependent patients found no reduction in use (62).

#### 4.3.3. Glu2/3 positive allosteric modulators.

Normalizing glutamate homeostasis reduces cocaine reinstatement in part by restoring glutamate tone on mGlu2/3 receptors (46, 63). Many other behavioral studies have shown that mGlu2/3 activation reduces psychostimulant seeking (33), and new mGlu2 PAMs may show greater drug versus natural reward selectivity (34). No clinical trials of mGlu2/3 PAMs in SUD have been conducted. However, for other neuropsychiatric disorders, promising preclinical results with these agents have not been matched by outcomes in clinical trials (64).

#### 4.3.4. mGlu5 negative allosteric modulators.

Many studies besides those described above have shown that mGlu5 NAMs reduce cocaine seeking (33, 34, 65), although there is concern that these agents can impair working memory (66). Interactions between mGlu5 NAMs and methamphetamine-induced behaviors are more complicated (34, 67, 68). Although most work in models of psychostimulant addiction has focused on reducing mGlu5 transmission, mGlu5 PAMs facilitate the extinction of drug memories (33). Development of mGlu5 PAMs biased toward specific pathways may improve their therapeutic profile (69). We note that work assessing mGlu5 as a target for SUD medications is mainly behavioral, rather than related to plasticity at excitatory synapses (the focus of this review). A notable exception concerns mGlu5/endocannabinoid-mediated LTD in NAcC MSNs, which is impaired after cocaine treatments ranging from a single intraperitoneal injection (70) to incubation of craving (Section 5.1). One might expect the impairment of this braking force on MSN activity to promote addiction-related behavior. However, examination of the related literature indicates a complex relationship between this synaptic depression and psychostimulant reward (see 67).

#### 4.3.5. Matrix metalloproteases.

Results described in Section 4.2 demonstrate the significance of constitutive and cue-evoked increases in MMP activity for multiple drugs of abuse. More broadly, there is growing interest in the therapeutic potential of MMP antagonists, especially if subtype-selective antagonists can be developed (71). This interest is based on preclinical studies establishing a critical role for MMPs in synaptic plasticity, combined with clinical evidence for therapeutic effects of nonselective MMP antagonists in brain disorders. Polymorphism variants and altered serum and brain levels of MMP-9 have been found in patients with SUD, and minocycline (which inhibits MMPs but also elicits many other effects) produced some benefit in clinical studies of SUD as well as other brain disorders. However, based on side effects observed after chronic administration of MMP antagonists and other considerations, MMP antagonists may be best suited to acute interventions.

## 5. ABSTINENCE MODELS AND THERAPEUTICALLY RELEVANT SYNAPTIC PLASTICITY

We focus on CP-AMPAR upregulation (see Section 2.1) during incubation of cocaine craving because of its potential for therapeutic targeting, describing work in NAcC and NAcS separately because of some intriguing apparent differences.

### 5.1. Plasticity in the Nucleus Accumbens Core During Forced Abstinence

In 2008, we showed that incubation of cocaine craving in rats is accompanied by persistent strengthening of glutamate synapses in NAcC MSNs through CP-AMPAR incorporation, as demonstrated by measuring the rectification index and sensitivity of AMPAR excitatory postsynaptic currents to the selective CP-AMPAR antagonist Naspm (16). After incubation, CP-AMPARs account for approximately 25% of overall AMPAR transmission in MSNs (16, 72). Most importantly, once CP-AMPARs are elevated, the expression of incubated cocaine seeking depends on their activation, as this seeking is inhibited by intra-NAcC infusion of Naspm (16) or internalization of CP-AMPARs via mGlu1 LTD (73, 74) (see the sidebar titled Cocaine Incubation Involves a Switch in Group I Metabotropic Glutamate Receptor–Mediated Long-Term Depression). CP-AMPAR upregulation is similarly required for methamphetamine incubation (75, 76).

There are two main subtypes of MSNs defined based on expression of D1 and D2 dopamine receptors and distinct anatomical projections. Originally conceptualized as “go” (D1 MSNs) and “no go” (D2 MSNs) pathways, it is now recognized that D1 and D2 MSNs interact in a complex manner to control motivated behavior; nonetheless, plasticity that increases excitatory drive to D1 MSNs generally promotes motivated behavior and is frequently observed after drug exposure (77–79). Consistent with this, CP-AMPARs upregulate exclusively in D1 MSNs of NAcC after cocaine incubation (80) and methamphetamine incubation (E.-K. Hwang & M.E. Wolf, unpublished data).

Intriguingly, the contribution of CP-AMPARs to synaptic transmission remains low (saline control levels) for several weeks after discontinuing cocaine self-administration; CP-AMPAR levels then rise quickly and remain high for months (72). During this delay, two adaptations occur that contribute to enabling CP-AMPAR upregulation.

The first adaptation involves mGlu1. Once CP-AMPARs have accumulated, pharmacologically induced mGlu1 LTD internalizes them (see above). However, there is also evidence across brain regions for an inverse relationship between endogenous glutamate tone at mGlu1 and CP-AMPAR levels (81). In the NAcC, a reduction in surface expression of mGlu1 precedes CP-AMPAR accumulation; restoring mGlu1 tone by administering repeated mGlu1 PAM injections prevents CP-AMPAR accumulation and incubation of cocaine craving (73). However, both CP-AMPARs and cocaine craving recover to incubated levels within days of discontinuing mGlu1 PAM injections, indicating suppression of incubation rather than its elimination. Methamphetamine incubation does not appear to involve reduced mGlu1 tone (82), although it is accompanied by elevated GluA1 translation (82) as found for cocaine (see Section 5.2).

The second adaptation related to CP-AMPAR accumulation during cocaine incubation involves the GluN3 subunit of the NMDAR, which confers atypical properties on NMDAR signaling (Section 2.2). GluN3-containing NMDARs do not contribute to NMDAR transmission in NAcC MSNs of drug-naïve rats. However, during early withdrawal from cocaine self-administration, NMDAR transmission is enhanced first by an increased contribution of GluN2B-containing NMDARs and shortly thereafter by GluN3-containing NMDARs; this enhancement persists for at least 2 months and GluN3 incorporation is required for incubation (83). Based on relationships between CP-AMPARs, GluN3-containing NMDARs, and mGlu1 discovered in VTA dopamine neurons (84, 85), it is possible that in NAcC, reduced mGlu1 surface expression (which occurs at approximately the same withdrawal time as synaptic incorporation of GluN3-containing NMDARs) triggers GluN3 plasticity, which in turn enables CP-AMPAR plasticity.

### 5.2. Homeostatic Plasticity May Explain Abstinence-Dependent Plasticity in the Nucleus Accumbens Core

A role for homeostatic plasticity in cocaine incubation is suggested by the delay in CP-AMPAR accumulation and by the absence of any obvious Hebbian stimulus during home-cage abstinence. Furthermore, CP-AMPAR upregulation during incubation occurs not only for psychostimulants but also for the opioid oxycodone (86), arguing against its initiation by specific actions of a drug class. Synaptic scaling is a form of homeostatic plasticity in which prolonged inactivity can lead to upscaling of AMPAR transmission while prolonged increases in activity can lead to downscaling, enabling network stability (87). Previously we proposed that strengthening of glutamate synapses during drug withdrawal represents upscaling, triggered by the contrast between high levels of neuronal activity during drug exposure and lower levels during abstinence (16, 88, 89). This would be adaptive during abstinence, when glutamate transmission is low, but maladaptive when a cocaine cue is presented, because AMPAR upregulation would enable a stronger synaptic and behavioral response to cue-evoked glutamate transmission.

One type of upscaling depends on retinoic acid (RA) signaling in dendrites. Normally, ongoing synaptic transmission maintains intracellular Ca^2+^ at levels sufficient to suppress RA synthesis. Prolonged blockade of neuronal activity, by reducing intracellular Ca^2+^, results in the disinhibition of RA synthesis and activation of the dendritic RA receptor RARα; this in turn leads to increased GluA1 translation and synaptic insertion of homomeric GluA1 CP-AMPARs (90). This cascade may be recapitulated in NAcC during cocaine incubation. Supporting this, ongoing protein translation in NAcC is required for maintenance of elevated CP-AMPARs (91) and expression of cocaine incubation (92), and translation of GluA1 (but not GluA2) is increased after cocaine incubation (93). Furthermore, cocaine incubation is accompanied by emergence of tonic RA signaling in NAcC, which is required for CP-AMPAR elevation and expression of incubation (80, 94). We hypothesize that the disinhibition of RA signaling results from the cumulative effect of adaptations occurring in early withdrawal. These adaptations may include reduced activity in brain regions sending glutamate inputs to NAc and in the NAc itself (e.g., 95, 96). The reduction in surface mGlu1 observed in early cocaine withdrawal may contribute because mGlu1 canonically signals through Ca^2+^ mobilization; furthermore, in cultured NAc neurons, decreasing mGlu1 tone increases surface CP-AMPARs via RA signaling and protein translation (97). The increase in GluN3-containing NMDARs (83) may also contribute since they are relatively impermeable to Ca^2+^ (Section 2.2) and may mediate the observed reduction in NMDAR-mediated Ca^2+^ entry into NAcC dendritic spines after cocaine incubation (83, 98).

### 5.3. Plasticity in the Nucleus Accumbens Shell During Forced Abstinence

Elegant work by Dong and colleagues (99, 100) has shown that cocaine self-administration in rats generates silent synapses in NAcS MSNs and that their maturation via AMPAR incorporation regulates incubation of cocaine craving. Their first study demonstrated CP-AMPAR upregulation through this mechanism in basolateral amygdala (BLA)-to-NAcS MSN synapses (38). This was evident as a progressive reduction in silent synapse levels during abstinence from cocaine self-administration that was mirrored by a progressive increase in CP-AMPAR levels. Detectable by abstinence day 10, the process was complete by day 45, when silent synapses had returned to control levels and CP-AMPARs were stably elevated (38). Eliminating CP-AMPARs from BLA-NAcS synapses, via an optogenetic protocol shown in a different pathway to depend on mGlu1 and NMDARs (101), re-silenced synapses and inhibited expression of incubated cocaine craving (38). Silent synapse generation and AMPAR filling were also demonstrated for other pathways during incubation of cocaine craving. Prelimbic medial prefrontal cortex (mPFC)-to-NAcC synapses matured by incorporating CI-AMPARs, which promoted incubation, while infralimbic mPFC-to-NAcS synapses matured by incorporating CP-AMPARs, which opposed incubation (101). Thus, there is heterogeneity in how cocaine-generated silent synapses mature (see also 102), although CP-AMPAR upregulation in the BLA-NAcS pathway seems dominant with regard to incubation since its reversal was sufficient to prevent the expression of incubation (see above). These and other findings (Section 5.4) establish silent synapses as a substrate for circuit remodeling related to storage of cocaine memories (103).

In the NAcS, homeostatic interactions between synaptic strength and membrane excitability are implicated in CP-AMPAR upregulation in rats during incubation of craving. The cascade is initiated when the new NMDARs associated with silent synapse generation send a false signal of increased excitatory synaptic transmission, triggering a reduction in membrane excitability; this in turn promotes a second round of homeostatic plasticity in which synaptic strength is increased via AMPAR insertion into silent synapses (104). Interestingly, synaptic changes and their timing in the NAcC during cocaine incubation do not match this NAcS cascade (see 83, 104). Furthermore, it is not known whether the membrane excitability of NAcC MSNs is reduced in the early stages of incubation. However, a cocaine self-administration regimen that in mice reduces MSN excitability in NAcS on withdrawal day 1 does not produce this effect in NAcC (105). Despite apparent subregion differences, the key point from the standpoint of CP-AMPAR-directed therapeutics is that reversing CP-AMPAR upregulation in either NAcC (Section 5.1) or NAcS is sufficient to prevent incubated cocaine craving.

Enhanced AMPAR transmission has also been found in NAcS MSNs after limited-access cocaine self-administration (106–108). In a study distinguishing D1 and D2 MSNs in mice, no plasticity was found in D2 MSNs, while D1 MSNs showed increased CP-AMPARs postsynaptic to infralimbic mPFC inputs and increased CI-AMPARs postsynaptic to ventral hippocampal inputs. An optogenetic LTD protocol (mGluR dependent) that normalized both pathways reduced cue-induced cocaine seeking, and this persisted after a week (107). The same group subsequently showed that a similar regimen did not elicit incubation, although they replicated selective CP-AMPAR upregulation in D1 MSNs. To reliably induce incubation in mice (which is more difficult than in rats), an extended-access and very high dose cocaine regimen was required, and this regimen produced pathway-specific CP-AMPAR upregulation in both D1 and D2 MSNs; it was speculated that D2 MSN involvement may relate to aversive effects of the high cocaine dose (108).

### 5.4. Plasticity Occurs in Nucleus Accumbens Shell Medium Spiny Neurons During a Cue-Induced Seeking Test

This plasticity was demonstrated in cocaine-incubated rats, in which cocaine-generated silent synapses in NAcS had already matured (i.e., recruited CP-AMPARs), through studies of post-retrieval memory destabilization and subsequent post-retrieval memory reconsolidation of cocaine-cue memories (109). Retrieval of cocaine memories upon a short cue re-exposure rapidly internalized CP-AMPARs, which were rerecruited to silent synapses by the end of the 6-h post-retrieval memory destabilization time window. If CP-AMPAR rerecruitment was prevented, incubated craving was reduced. The same interference manipulations performed outside the destabilization window did not influence incubation, indicating that once silent synapses have re-matured, the cocaine-cue memory is no longer vulnerable (109). These results suggest that internalization and rerecruitment of CP-AMPARs at cocaine-generated synapses are two cellular components of the retrieval-induced destabilization and reconsolidation of incubation-related memories, with the dynamics of CP-AMPARs dictating the state of these memories—either destabilized or reconsolidated. These results also indicate that reconsolidation might be attenuated if a relapse event occurs while a CP-AMPAR-targeting therapy is on board (Section 5.5). Notably, the transient depression of AMPAR transmission in NAcS MSNs during cue-induced cocaine seeking in abstinence that is detailed above contrasts with the transient potentiation reported in NAcC D1 MSNs during cue-induced cocaine seeking after extinction or abstinence (Section 4.2). These opposite responses may reflect core-shell differences or occur in distinct neuronal ensembles.

### 5.5. Targets for Therapeutic Intervention Revealed by Studying Plasticity During Abstinence

The demonstration of incubation of craving in humans (see the sidebar titled Incubation of Craving in Humans) underscores the translational potential of therapeutic targets revealed by rodent research on incubation. As detailed above, CP-AMPAR upregulation in NAc drives psychostimulant seeking after protracted abstinence (Sections 5.1–5.4) and perhaps after extinction (Section 4.1). Cocaine also upregulates CP-AMPARs in VTA (20) and PFC (110). In opioid models, upregulation of CP-AMPARs in NAc MSNs occurs after experimenter-administered and self-administered opioids (86, 111–113), is required for oxycodone incubation (86), contributes to morphine and fentanyl CPP (55, 111), and mediates aversive effects of morphine withdrawal (112). Alcohol exposure can also increase CP-AMPARs in NAc (114, 115), and one study showed that CP-AMPAR upregulation in NAcC-projecting BLA neurons supports operant alcohol self-administration (116). This section therefore focuses on approaches to normalize this broadly significant plasticity.

#### 5.5.1. mGlu1 positive allosteric modulators.

Repeated administration of a systemically active mGlu1 PAM in early abstinence delays cocaine-induced CP-AMPAR upregulation in NAcC and incubation (Section 5.1) (73), potentially providing a window of opportunity for behavioral interventions. After protracted abstinence, eliciting mGlu1 LTD with an mGlu1 PAM reverses CP-AMPAR upregulation and decreases cocaine craving, facilitating continued abstinence (see the sidebar titled Cocaine Incubation Involves a Switch in Group I Metabotropic Glutamate Receptor–Mediated Long-Term Depression). Chronic administration of mGlu1 PAMs may be feasible, as a substantial body of preclinical work indicates very low potential for adverse effects. Seizures, the major liability originally associated with Group I mGluR stimulation, are now recognized as being mediated by mGlu5 (117). mGlu1 PAMS do not affect locomotion (118–121), except at a very high dose (122), or motivation for natural rewards (73, 122). Despite these encouraging results, off-target effects could occur at synapses enriched in CP-AMPARs under drug-naïve conditions, such as those on interneurons (123, 124). Finally, several studies, mostly using intracranial injections, found that mGlu1 NAMs reduce reinstatement of cocaine seeking (34). It is possible that the regimens used or the regions targeted were not associated with CP-AMPAR upregulation, so distinct effects of mGlu1 modulation occurred. No such effects have been reported in abstinence models. Interestingly, mGlu1 or mGlu5 PAMs acting in mPFC might correct a deficit in extinction learning that promotes incubation (125).

#### 5.5.2. Retinoic acid–mediated homeostatic plasticity.

RA signaling in NAcC through dendritic RARα may underlie the incubation of cocaine craving (Section 5.2). Therefore, RARα antagonists, already under development as therapeutics for another indication (126), are potential anti-craving drugs. In another interesting connection between RA and addiction, two transcriptomics studies linked RA signaling in the NAc with the interaction between environmental enrichment (EE) and cocaine self-administration (127, 128). EE is a behavioral intervention that attenuates drug seeking (Section 5.5.5). It remains to be determined how these transcriptomic results, which presumably reflect canonical effects of nuclear RARs on transcription, are related to results linking incubation to homeostatic plasticity mediated by dendritic RARα.

#### 5.5.3. Acid-sensing ion channels.

Protons (hydrogen ions) are released from neurotransmitter vesicles (including glutamate vesicles) during neurotransmission and activate a family of ionotropic acid-sensing ion channels (ASICs) on dendritic spines, with resultant currents accounting for approximately 5% of the excitatory synaptic current in NAc MSNs (129). Disruption of ASIC1A in NAcC leads to synaptic changes that mimic those reported after cocaine withdrawal, including increased CP-AMPAR levels (129, 130). Conversely, potentiating ASIC1A function by disrupting carbonic anhydrase 4 reduced cue-induced cocaine seeking and reduced the AMPA/NMDA ratio in NAcC MSNs (131). Thus, manipulating ASIC1A function by inhibiting carbonic anhydrase 4 (using the FDA-approved inhibitor acetazolamide) might reduce craving during abstinence.

#### 5.5.4. Sleep.

Sleep abnormalities accompany SUD, and improving sleep quality may be beneficial (132). In rats, incubation of craving is accompanied by changes in sleep patterns, including reduced number and duration of REM episodes during the light phase (when most sleep occurs). Increasing REM sleep time and continuity during the light phase attenuated incubation of cocaine craving and accompanying CP-AMPAR elevation in NAcS MSNs. Conversely, treatment eliciting sleep fragmentation accelerated incubation and CP-AMPAR accumulation (133, 134). These effects were shown to involve changes in the activity of melanin-concentrating hormone neurons in the lateral hypothalamus, which project to both REM regulatory regions and reward circuitry, including NAcS MSNs (134, 135). Given that CP-AMPAR plasticity appears to be downstream of cocaine-induced sleep disruption, these results underscore the potential of CP-AMPAR-directed therapeutic approaches.

#### 5.5.5. Environmental enrichment.

Housing rats under conditions of EE after drug self-administration decreases cue-induced responding after 2–3 weeks of abstinence across drug classes (e.g., 136). Focusing on cocaine incubation, one study found that EE reduced seeking throughout abstinence but did not abolish incubation (137), while another (using conditions that promote more robust incubation) found attenuation of incubation during EE but dissipation of this effect when EE was discontinued (138). Interestingly, by harnessing synaptic plasticity, effects of EE can be made longer lasting. As described in Section 5.3, optogenetic LTD removes CP-AMPARs from BLA-NAcS synapses and thereby re-silences these synapses, but CP-AMPARs recover within 24 h, leading to recovery of incubated craving. However, if EE is introduced during the window of CP-AMPAR absence, silent synapses re-mature by incorporating CI-AMPARs (rather than CP-AMPARs), returning this pathway to a state resembling that of control animals. Subsequently, incubation is persistently suppressed (139).

## 6. CONCLUSIONS

Synaptic plasticity research has established potentiation of excitatory synapses in the NAc as a critical mediator of psychostimulant seeking and has helped identify pharmacological and behavioral approaches to reverse this plasticity and reduce drug seeking. Of the first-generation plasticity-based approaches (identified over 10 years ago; Section 4.3), only *N*-acetylcysteine has advanced to clinical trials for cocaine use disorder (Section 4.3.2). While some results were promising, they fell short of preclinical findings. Of course, there are many possible explanations for this. Most relevant to this review, while animal studies can isolate cue-induced craving from other variables, this is only one of many factors contributing to relapse vulnerability in humans. Furthermore, patients in clinical trials are heterogeneous, and polysubstance abuse is common. Nonetheless, second-generation plasticity-based approaches (Section 5.5) warrant evaluation. mGlu1 PAMs are particularly promising as they directly reverse CP-AMPAR upregulation—which promotes addiction-related behaviors across drug classes, drug regimens, and brain areas—and through this mechanism may synergize with some behavioral interventions.

**Figure 1:**
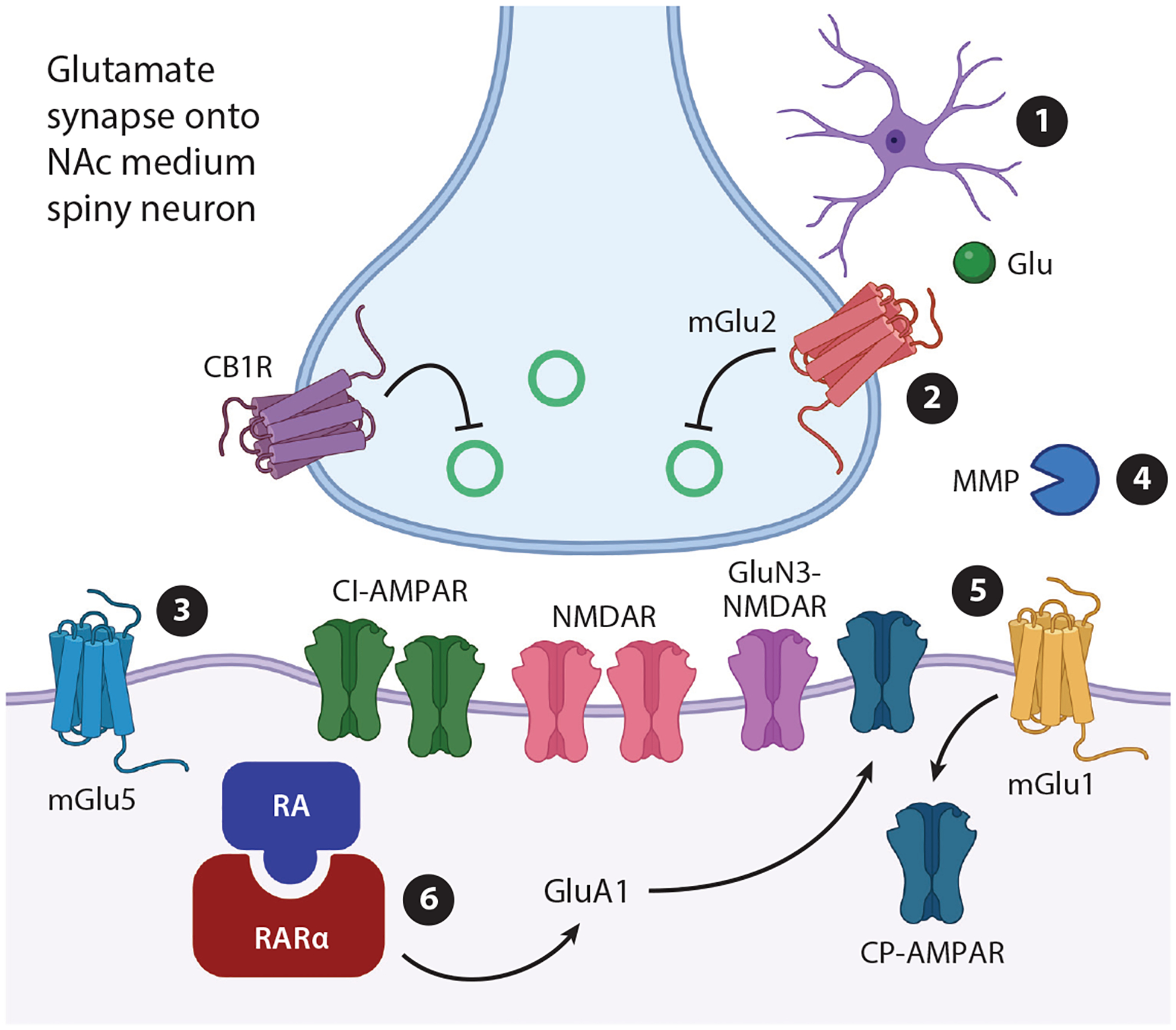
Potential targets for anti-craving therapeutics discovered by studying psychostimulant-induced synaptic plasticity in animal models of addiction. Targets 1–4 were identified mainly through extinction-reinstatement studies (Section 4). Targets 5 and 6 were identified mainly through abstinence studies (Section 5). (①) Cocaine disrupts glutamate homeostasis via reduced function of the astrocytic cystine-glutamate exchanger and glutamate transporter GLT-1; these effects are normalized by *N*-acetylcysteine and ceftriaxone through a cascade described in Sections 4.1 and 4.2. The purple cell represents an astrocyte. (②) mGlu2 or mGlu2/3 positive allosteric modulators (PAMs) reduce glutamate release and thereby reduce glutamate-driven psychostimulant seeking. (③) Glutamate spillover resulting from cocaine-induced disruption of glutamate homeostasis leads to excessive activation of AMPA receptors (AMPARs) and mGlu5; the latter can be reduced by mGlu5 negative allosteric modulators (see text for discussion of AMPARs). (④) Glutamate spillover also activates matrix metalloproteinases (MMPs), which remodel the extracellular matrix to enable plasticity associated with reinstatement of cocaine seeking; this could be opposed using an MMP antagonist. (⑤) While Ca^2+^-impermeable AMPARs (CI-AMPARs) mediate excitatory synaptic transmission in the NAc of drug-naïve rats, upregulation of Ca^2+^-permeable AMPARs (CP-AMPARs) mediates the expression of incubated cocaine and methamphetamine seeking; this can be reversed by mGlu1 PAMs through mGlu1 long-term depression expressed via CP-AMPAR internalization. (⑥) Retinoic acid (RA) signaling through its dendritic receptor RARα underlies homeostatic plasticity that contributes to CP-AMPAR accumulation and incubation of craving, suggesting the potential utility of RARα antagonists. Figure adapted from images created with BioRender.com.

Compared to agents in the pipeline that are designed to block rewarding effects of cocaine and methamphetamine, plasticity-based therapeutics would offer advantages because they target neuroadaptations that maintain persistent relapse vulnerability even in the absence of active drug use. Is there cause for optimism regarding their implementation? On the one hand, druggable targets have been identified (Figure 1). On the other, developing compounds with acceptable therapeutic profiles and advancing through clinical trials is notoriously difficult. Yet, this seems a battle well worth fighting. The present standard of care for cocaine use disorder is behavioral intervention (motivational interviews, contingency management, community reinforcement and cognitive behavioral therapy) (3). Despite the efficacy of these approaches, there are barriers related to cost, accessibility of providers, coordination with other care, and social factors. Plasticity-based therapeutics may assist patients in maintaining abstinence despite these challenges.

## INCUBATION OF CRAVING IN HUMANS

Incubation has been demonstrated in humans during abstinence from cocaine (145), methamphetamine (146), nicotine (147) and alcohol (148). A recent review argued that incubation of craving does not occur during opioid abstinence based on studies comparing measures of craving after different periods of abstinence (149). However, nearly all of the studies included performed their early abstinence assessment after the onset of incubation as predicted from rats, and in many cases patients were receiving methadone or participating in interventional studies where baseline craving was indexed to onset of the intervention, not onset of abstinence. Thus, more work is required to assess whether incubation of opioid craving occurs in humans.

## COCAINE INCUBATION INVOLVES A SWITCH IN GROUP I METABOTROPIC GLUTAMATE RECEPTOR-MEDIATED LONG-TERM DEPRESSION

In NAcC MSNs of drug-naïve animals, group I mGluR activation elicits synaptic depression that depends on mGlu5 activation and is expressed presynaptically via endocannabinoid-mediated reduction of glutamate release; mGlu1 antagonists do not influence this synaptic depression. After incubation, this mGlu5-dependent mechanism is replaced by mGlu1 LTD expressed via CP-AMPAR internalization (150). Eliciting mGlu1 LTD with mGlu1 PAMs prevents expression of incubated cocaine or methamphetamine seeking (73, 75) and reverses cocaine-induced CP-AMPAR upregulation in VTA and prefrontal cortex (20, 110). Synapses in other brain regions with high levels of CP-AMPARs also express this mGlu1 LTD (81).

## SUMMARY POINTS

1. Synaptic plasticity, especially potentiation of AMPA receptor (AMPAR) transmission in the nucleus accumbens (NAc), underlies psychostimulant craving and relapse-like behavior in animal models.
2. Pharmacological approaches that reverse psychostimulant-induced plasticity and thereby reduce drug seeking have been identified, yielding some potentially druggable targets.
3. Synaptic plasticity studies have also identified strategies to increase the efficacy of behavioral interventions in animal models.
4. Ca^2+^-permeable AMPAR upregulation is strongly linked to psychostimulant seeking, so treatments that reverse this plasticity (e.g., systemically administered mGlu1 positive allosteric modulators) hold particular promise.

## FUTURE ISSUES

1. A major unanswered question is the extent to which drug-induced plasticity in animal models resembles that occurring in humans, although strategies to assess plasticity in the human brain are gaining traction (140, 141).
2. There is tremendous interest in transmagnetic stimulation and particularly deep brain stimulation (which can access deeper structures such as the NAc) for the treatment of psychiatric disorders, including substance use disorder (SUD); synaptic plasticity research will inform the refinement of these approaches (142, 143).
3. The *N*-methyl-d-aspartate receptor antagonist ketamine elicits rapid antidepressant effects in part by producing a rapid “reset” of dendritic protein translation and synaptic properties; such effects may have potential for normalizing synaptic function in SUD as well as other disorders (144). Psychedelics may also have therapeutic potential, but little is known about their effects at the level of excitatory synaptic plasticity.

## Abbreviations

Synaptic plasticity: the ability of synapses to change their weight or connectivity in response to specific experience
Medium spiny neurons (MSNs): GABAergic principal neurons that integrate glutamate inputs from cortical and limbic regions; their output to motor-related circuitry drives motivated behavior
Drug self-administration: animals perform an operant response (lever-press or nose-poke) to deliver intravenous drug infusions, often paired with a cue or context
Ionotropic glutamate receptors: glutamate-gated ion channels named for agonists that preferentially activate them, namely AMPA, NMDA, and kainate
Ca^2+^-impermeable AMPARs (CI-AMPARs): GluA2-containing AMPARs in which GluA2 subunits have undergone RNA editing, rendering the receptor channel impermeable to Ca^2+^
Ca^2+^-permeable AMPARs (CP-AMPARs): AMPARs lacking GluA2 or sometimes containing unedited GluA2; besides Ca^2+^ permeability, they exhibit higher conductance and other differences from CI-AMPARs
Rectification index: electrophysiological measure of the contribution of CP-AMPARs to synaptic transmission based on inward rectification of CP-AMPAR- but not CI-AMPAR-mediated currents
GluN3 subunit: inclusion of this subunit endows NMDARs with lower conductance, reduced Ca^2+^ permeability, and insensitivity to Mg^2+^ block
Limited-access regimen: drug is available for 1–2 h/session for 1–2 weeks; such regimens are not thought to model compulsive drug seeking
Extinction training: after establishing stable drug self-administration, animals undergo repeated sessions during which the operant response no longer results in drug delivery
Reinstatement test: conducted after extinction training, assesses ability of a drug-associated cue/context, stressor, or drug injection to elicit resumption of drug seeking
Extended-access regimen: employs longer sessions (6 h/session for 10–20 days) or longer duration (2 h/session for 1–2 months), better modeling compulsive use
Cue-induced seeking test: test performed after abstinence (but without extinction training); operant responses elicit delivery of the previously drug-paired cue but no drug
Voluntary abstinence models: abstinence is maintained by imposing a mutually exclusive choice between drug and non-drug rewards or by pairing drug with punishment
AMPA/NMDA ratio: electrophysiological measure of relative weights of AMPARs versus NMDARs; increases suggest greater AMPAR transmission (if NMDARs are confirmed to be constant)
Conditioned place preference (CPP): procedure to assess rewarding effects of drugs by measuring the time animals spend in an environment previously paired with drug administration
Silent synapses: contain NMDARs but lack functionally stable AMPARs and serve as substrates for forming new or remodeling existing circuits
Post-retrieval memory destabilization: following retrieval, memories are temporarily destabilized and vulnerable to elimination for several hours, until reconsolidation occurs
Post-retrieval memory reconsolidation: by the end of the destabilization time window, memories are restabilized and stored, becoming resistant to disruption again
